# Large Scale Aggregate Microarray Analysis Reveals Three Distinct Molecular Subclasses of Human Preeclampsia

**DOI:** 10.1371/journal.pone.0116508

**Published:** 2015-02-13

**Authors:** Katherine Leavey, Shannon A. Bainbridge, Brian J. Cox

**Affiliations:** 1 Department of Physiology, University of Toronto, Toronto, Ontario, Canada; 2 Interdisciplinary School of Health Sciences, University of Ottawa, Ottawa, Ontario, Canada; Center for Human Reproduction, UNITED STATES

## Abstract

**Background:**

Preeclampsia (PE) is a life-threatening hypertensive pathology of pregnancy affecting 3–5% of all pregnancies. To date, PE has no cure, early detection markers, or effective treatments short of the removal of what is thought to be the causative organ, the placenta, which may necessitate a preterm delivery. Additionally, numerous small placental microarray studies attempting to identify “PE-specific” genes have yielded inconsistent results. We therefore hypothesize that preeclampsia is a multifactorial disease encompassing several pathology subclasses, and that large cohort placental gene expression analysis will reveal these groups.

**Results:**

To address our hypothesis, we utilized known bioinformatic methods to aggregate 7 microarray data sets across multiple platforms in order to generate a large data set of 173 patient samples, including 77 with preeclampsia. Unsupervised clustering of these patient samples revealed three distinct molecular subclasses of PE. This included a “canonical” PE subclass demonstrating elevated expression of known PE markers and genes associated with poor oxygenation and increased secretion, as well as two other subclasses potentially representing a poor maternal response to pregnancy and an immunological presentation of preeclampsia.

**Conclusion:**

Our analysis sheds new light on the heterogeneity of PE patients, and offers up additional avenues for future investigation. Hopefully, our subclassification of preeclampsia based on molecular diversity will finally lead to the development of robust diagnostics and patient-based treatments for this disorder.

## Background

Preeclampsia (PE) is a multi-system disorder of pregnancy defined by the onset of maternal hypertension and proteinuria in the latter half of gestation. This pathology affects 3–5% of all pregnancies and is responsible for 63,000 maternal deaths worldwide each year [1]. To date, PE has no cure short of the removal of the causative organ, the placenta, which may necessitate a preterm delivery and result in both acute and chronic health risks to the child. The incidence of PE has raised relentlessly [2] and effective screening tools and/or treatments have yet to be discovered. While a correlation is observed between elevated levels of various placental proteins in maternal blood serum (ex. sFLT1, sENG and PGF) in early pregnancy and the prediction of future PE development [3], the false negative detection rates are too high for clinical use [4]. Additionally, the employment of clinical biometrics, as well as Doppler ultrasound measurements, typically yield similar results [5].

These challenges have led researchers to apply genome-wide profiling techniques, such as microarray analysis, in cases of PE in order to better understand the etiology of placental dysfunction in this disorder. The primary anticipated outcome of all microarray studies performed to date was the identification of differentially expressed genes in the PE placentae, as a cohesive group, compared to a control group. However, in the largest study performed [6] (N = 23 PE patients), considerable variability was observed and ~80% of the gene expression variance in the data set could not be explained by the binary clinical classification of “control” versus “PE” and other covariates. A similar observation is also inferred from two recent meta-analyses of PE placental microarray data sets that found few significant genes in common [7,8]. This has led us [9] and others [10–12] to hypothesize that preeclampsia is a spectral disorder driven by the deregulation of different molecular pathways.

Previous large-scale microarray analysis (N>70) in other multi-factorial, heterogeneous diseases, such as cancer [13,14], has been very beneficial for discovering molecular subclassifications of patients. Furthermore, statistical methods now exist to merge smaller microarray data sets into larger aggregate data sets [15]. These methods utilize normalization of data ranges and batch correction to enable the comparison of gene expression values across different platforms. We therefore chose to test our hypothesis using a bioinformatics approach of aggregating several previously published placental microarray data sets into a single large data set with sufficient sample size to identify PE subclasses. This was done using unsupervised clustering techniques, which blindly group samples without knowledge of their pathology status, and is fundamentally different from a meta-analysis where each study is assigned a weight and an average is taken over all studies to identify genes with significance. The outcome of our analysis will be the molecular-based classification of patients into distinct pathology groups, leading to improved patient categorization prior to treatment or clinical trial inclusion.

## Results

### Assembly of the aggregate data set

Our literature search identified 38 previously published microarray studies examining gene expression within the PE placenta (as of March 2013), seven of which were found to meet our inclusion and exclusion criteria [6,16–21] (see Methods) (Table 1). Importantly, these studies spanned several geographic regions including Asia, Europe and North America. As these studies utilized different array platforms and sample preparation methods, they could not be directly compared. We therefore selected an empirical Bayes method of normalization and batch correction to combine the seven selected data sets into a virtual microarray of genes with probes found on all array platforms used in the original studies. After merging, the aggregate data set contained 173 samples (77 PE and 96 controls) with expression values for 14,653 genes, making this the largest data set of PE samples ever assembled. In genome wide expression analyses, most genes are invariable across the samples. These genes are usually removed by subjectively selecting those with highly variable differences between the known treatment groups. This introduces a bias in the genes in favour of those differentially expressed between phenotypes and prevents the discovery of novel subgroups. To avoid this, we removed invariant genes using an unbiased filtering for those with expression variance in the top quartile, reducing the number of genes utilized for sample clustering to 3,663.

**Table 1:** The 7 previously published PE-associated placental microarray studies found to meet our inclusion and exclusion criteria (see Methods).

GEO ID	Platform	PE	Controls	Total Samples
GSE30186	Illumina HumanHT-12 V4.0 expression beadchip	6	6	12
GSE10588	AGI Human Genome Survey Microarray Version 2	17	26	43
GSE24129	Affymetrix Human Gene 1.0 ST Array	8	8	16
GSE25906	Illumina human-6 v2.0 expression beadchip	23	37	60
GSE43942	NimbleGen Homo sapiens HG18 090828 opt expr HX12	5	7	12
GSE4707	Agilent-012391 Whole Human Genome Oligo Microarray G4112A	10	4	14
GSE44711	Illumina HumanHT-12 V4.0 expression beadchip	8	8	16
**TOTAL:**		**77**	**96**	**173**

### Clustering and covariate analysis

**Fig 1:**
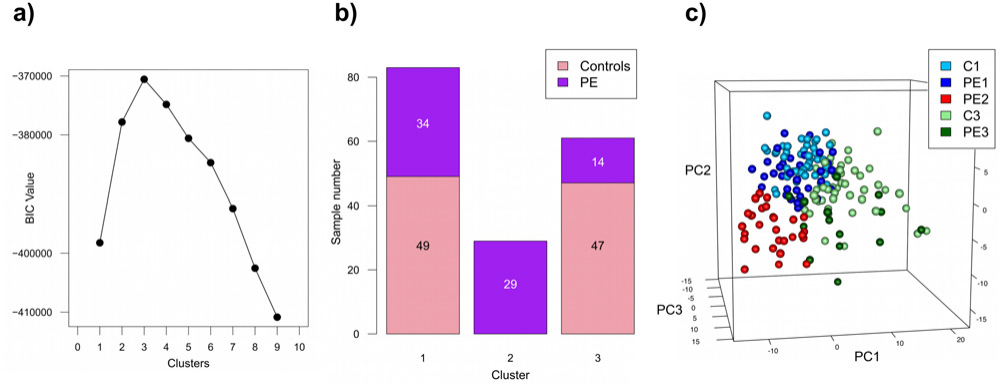
Unsupervised multivariate model-based clustering of the aggregate data set of 77 preeclamptics and 96 controls. (A) The Mclust model VEI (diagonal, equal shape) gave the best performance based on the Bayesian Information Criterion (BIC; y-axis) and an optimal cluster number of 3 was selected (clusters; x-axis). (B) Cluster 2 was composed entirely of PE samples while the remaining two clusters consisted of a mixture of preeclamptic and control samples. (C) Principal component analysis (PCA) was performed on the data to allow for cluster visualization in component space. Under PCA, samples closer together demonstrate higher similarity in gene expression. PC1–3 are principal components 1–3, respectively, while colours indicate cluster membership (1, Blue; 2, Red; 3, Green), with light shades denoting controls and dark shades indicating preeclamptics.

The combined set of PE samples and controls was treated as a single large data set and analyzed by unsupervised multivariate model-based clustering. This method employs the Bayesian Information Criterion to allow for the identification of an optimal number of sample clusters based solely on observed patterns of gene expression, independent of official clinical diagnosis, while preventing over fitting or under fitting of the data. In this case, clustering with the optimal model (VEI: diagonal, equal shape) revealed three distinct molecular groups of placental gene expression (Fig. 1a). Significantly, cluster 2 was composed entirely of preeclamptic patients (Fig. 1b, c). Surprisingly, the controls split between clusters 1 and 3, and each of these control subclasses co-clustered with PE samples, indicating the existence of at least three subclasses of preeclampsia.

**Fig 2:**
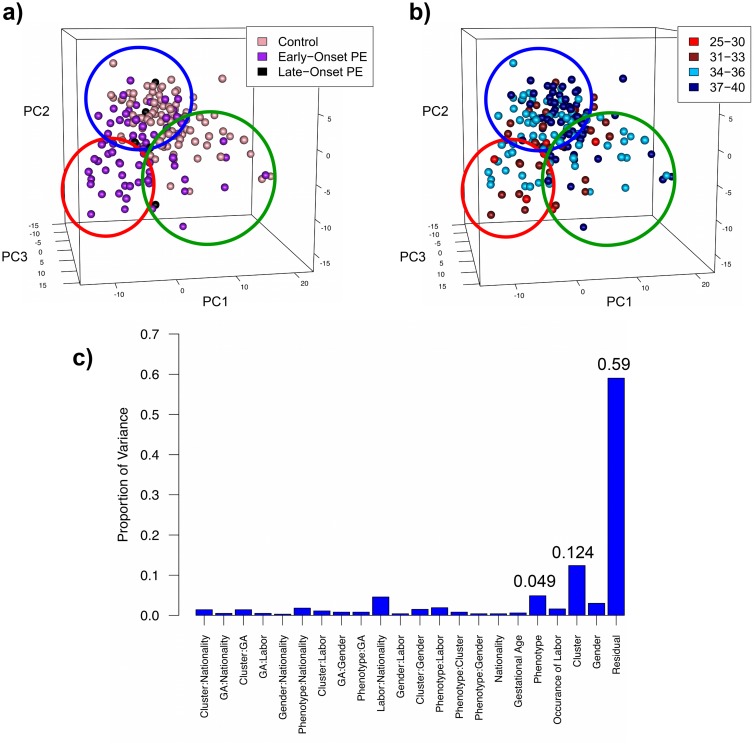
Potential confounding factors of clustering. (A) No differential segregation of late-onset PE samples was observed compared to the remaining early-onset preeclamptics. Molecular cluster members are identified by color-coded circles (cluster 1—blue, cluster 2—red, cluster 3—green). (B) The few identified preterm controls (<34 weeks) were found in cluster 3 (circled in green). The youngest identified PE samples (<30 weeks) were in cluster 2 (circled in red) while the oldest PE samples (>37 weeks) belonged to cluster 1 (circled in blue). (C) Principal variance component analysis (PVCA) on the full data set of preeclamptics and controls was performed to quantify the effect of each factor (and pairwise interactions between factors) on the gene expression variability within the data set. Minimal contributions were observed from the covariates and most pairwise interactions. Importantly, however, cluster membership was found to be responsible for more than twice the transcriptional variation than the clinical diagnosis (12.4% versus 4.9%), indicating a diversity of molecular groups with common clinical presentation. The residual variability observed (59%) was likely due to additional covariates that could not be accounted for as well as underlying non-pathological heterogeneity amongst the human samples. Although this value is still high, it is significantly reduced compared to a previously published PVCA interrogation of placental gene expression (residual: 86%) [6], employing a binary clinical classification.

Differences in covariates may explain these unexpected results, as it has been reported that the occurrence of labor and fetal sex may alter placental gene expression [22,23]. We observed no associations between cluster membership and nationality, occurrence of labor, original study membership, or fetal sex, as statistically supported by chi-squared analysis (Table 2, S1 Fig.). This is an important finding as it indicates that our observation of considerable heterogeneity amongst PE samples is a global phenomenon evident in all data sets and is independent of fetal sex or labor. Additionally, although the aggregated data set contained several known late-onset PE samples, there was no differential segregation of these patients compared to the remaining early-onset preeclamptics (Fig. 2a). We did note a modest correlation between gestational age (GA) and cluster membership, with younger samples generally gravitating towards clusters 2 and 3, and older samples often found in cluster 1 (Fig. 2b). To better understand the effect of covariates and our novel subgroups on gene expression, we subjected the full set of preeclamptic and control samples to principal variance component analysis (PVCA). This analysis indicated that the covariates were responsible for very little of the transcriptional variation within the data (Fig. 2c), supporting our chi-squared results. The exception was cluster membership, which was found to account for more than twice the variability of gene expression in the samples than the phenotypes of PE and control (12.4% versus 4.9%). This indicates that cluster membership better explains differences in gene expression among the samples than binary clinical classification. Overall, the unsupervised clustering, chi-squared and PVCA results all support the existence of multiple distinct molecular forms of PE, as well as indicate the possibility of two subclasses of controls.

**Table 2:** Potential effect of covariates on cluster membership by chi-squared analysis.

Variable	Chi-squared	df	P-value
Phenotype^a^	43.464	2	3.647e-10
Original Study^b^	10.381	12	0.5826
Fetal Sex^c^	2.550	2	0.2794
Nationality^d^	4.202	8	0.8385
Gestational Age^e^	21.409	6	0.0015
Occurrence of Labor^f^	1.597	4	0.8094

^a^ PE or control

^b^ GSE30186, GSE10588, GSE24129, GSE25906, GSE43942, GSE4707, or GSE44711

^c^ Male or female (predicted based on the expression of two Y-chromosome genes: UTY and USP9Y)

^d^ Canada, China, Finland, Japan, or USA

^e^ As bins of 25–30 weeks, 31–33 weeks, 34–36 weeks, or 37–40 weeks

^f^ Yes, no, or unknown.

### Investigation into the splitting of the control samples

**Fig 3:**
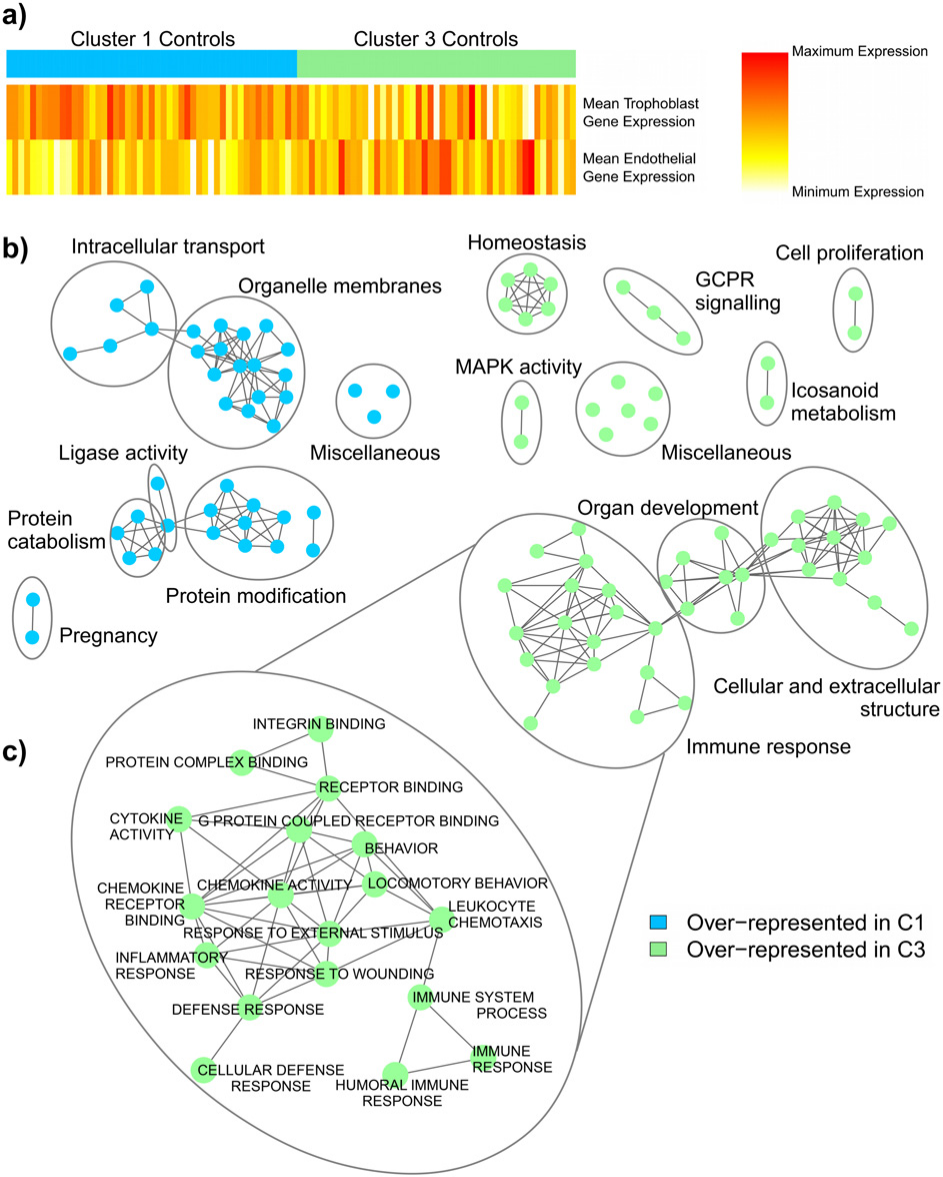
Investigation into the splitting of the control samples. (A) The possible existence of a sampling bias was explored using a heatmap of the mean expression of 35 known endothelial-enriched genes and the mean expression of 20 known trophoblast-enriched genes. Samples with high gene expression are coloured red, with a gradient of decreasing expression down to white. We observed a general up-regulation of trophoblast marker expression (top panel) in cluster 1 controls (blue), and an increased expression of endothelial genes (bottom panel) in controls belonging to cluster 3 (green), implying that a mild sampling bias may be involved in the formation of the two control subclasses. A heatmap with the expression pattern of each individual gene can be found in S2 Fig. (B) The controls in clusters 1 and 3 were compared by gene-set enrichment analysis (GSEA). Results were visualized in Cytoscape and networks of related ontologies (shown as coloured nodes connected by grey edges, representing common genes between gene sets) were circled and assigned a group label. Ontologies labeled as “miscellaneous” did not share genes with any of the networks. Cluster 1 controls (C1) revealed a significant over-representation of genes generally involved in pregnancy and normal pregnancy processes (blue), while cluster 3 controls (C3) demonstrated an increase in genes related to organ development and extracellular matrix structure (green), as well as an abundance of terms associated with immune response. (C) Enlargement of the immune response network enriched to cluster 3 controls with individual gene sets labelled. Therefore, the controls are most likely splitting because the placentas found in cluster 1 were involved in fairly “normal” pregnancies, while those belonging to cluster 3 experienced a strong immunological response during gestation, significantly affecting their gene expression.

To determine why the control samples split into two clusters, we first tested for sampling bias in the placental biopsies themselves. The placenta is not a homogeneous structure and single samples may not accurately reflect the mean gene expression of the tissue [24,25]. Given that several of the included studies reported the collection of a single placental villus biopsy per patient, this could have led to the spurious splitting of the controls into clusters 1 and 3. Based on sets of genes previously established as enriched to either trophoblast or endothelial cells [9] (S1 Table), we observed a general up-regulation of trophoblast marker expression in cluster 1 controls, compared to an increased expression of endothelial genes in controls belonging to cluster 3 (Fig. 3a, S2 Fig.). This was also consistent with a statistically significant difference in the expression of GCM1, a known regulator of vascular formation in mouse and human placentas [26,27], between cluster 1 and cluster 3 controls (8.81 versus 8.52, p-value < 0.01). We concluded that a mild sampling bias may be involved in the formation of the two control subclasses, although the observed difference in gestational age between clusters 1 and 3 (Fig. 2b) could also explain this disparity in the proportions of trophoblast and endothelial cells.

As sample bias seems to have played only a minor role in driving the control samples into two clusters, we next tested if these non-preeclamptic samples demonstrated underlying physiological or pathological differences. To assess this, we compared the controls in clusters 1 and 3 by gene-set enrichment analysis (GSEA). By assembling genes together into functional pathways, GSEA is capable of identifying potentially important collections of molecular changes (gene sets) that affect each group. This investigation revealed an over-representation of genes generally involved in reproduction and pregnancy in cluster 1 controls, along with genes associated with normal pregnancy processes such as intracellular transport, organelle function, and protein modification and activity (Fig. 3b, S2 Table). In contrast, cluster 3 controls demonstrated an abundance of genes involved in specific signalling and metabolic pathways, as well as terms related to homeostasis, organ development, and extracellular matrix structure **(** Fig. 3b, S3 Table). However, the most surprising finding was a significant enrichment of immune response terms to cluster 3 controls, including inflammatory response, defense response, cytokine activity, and response to wounding. Further investigation into this over-representation of immune terms revealed an enrichment of genes associated with graft-versus-host disease and allograft rejection in cluster 3 controls, many of which belong to HLA class II (S3 Table). These results therefore indicate that the controls split into two subclasses due to an underlying pathology difference: cluster 1 controls had fairly “normal” pregnancies, while cluster 3 samples experienced a strong immunological response, which significantly affected their placental gene expression, despite successfully avoiding the development preeclampsia.

### Assessment of known PE markers

**Fig 4:**
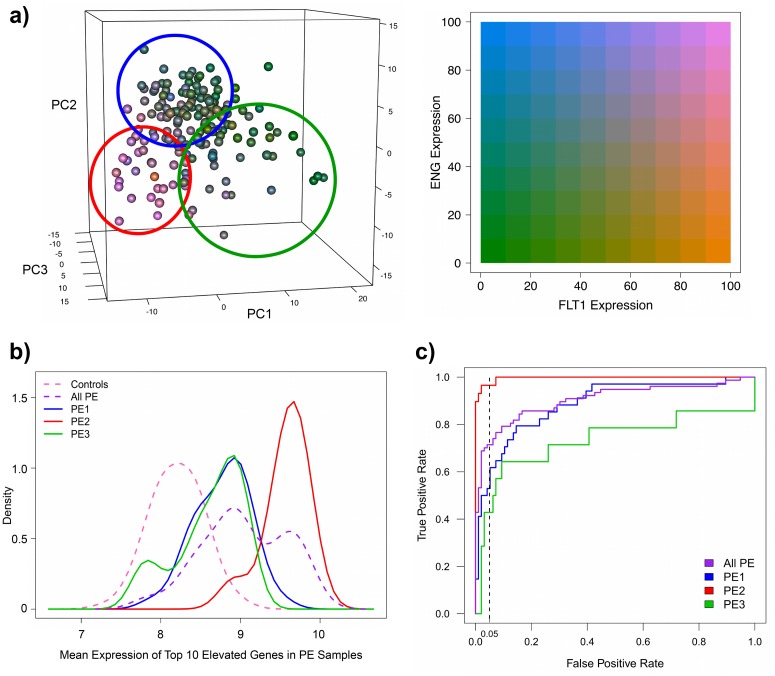
Biomarkers of preeclampsia. (A) Only the samples in the PE-enriched cluster 2 (circled in red) demonstrated increased expression of the two most frequently studied markers of PE, sFLT1 and sENG (pink), while the remaining preeclamptics in clusters 1 (circled in blue) and 3 (circled in green) displayed low levels of both of these markers (green), in line with control values of expression. (B) Density plots of the mean expression of the top 10 genes significantly elevated in the preeclamptics compared to the controls (LEP, HTRA4, FSTL3, LHB, TREM1, ENG, PAPPA2, FLT1, INHBA, and INHA). Considerable overlap in expression was observed between the controls (dashed pink) and the preeclamptics as a cohesive group (dashed purple). However, when the preeclamptic placentas were split into their three subclasses, cluster 2 PE samples (PE2; solid red) were easily separated from the controls, while the preeclamptics in clusters 1 (PE1; solid blue) and 3 (PE3; solid green) still demonstrated considerable overlap. (C) Naive Bayes classification using these 10 PE markers was able to distinguish >95% of the PE samples in cluster 2 (PE2; red) from the controls at a 5% false positive rate (dashed black line), while only ~50% and ~40% of the preeclamptics in clusters 1 (PE1; blue) and 3 (PE3; green), respectively, could be correctly categorized. This led to an overall ability of these markers to correctly identify approximately 70% of all the PE samples as preeclamptic (purple), as has been published. This analysis indicates that poor biomarker performance is likely due to molecular heterogeneity resulting from different etiological origins of preeclampsia.

There is great interest in the identification of biomarkers to predict preeclampsia prior to the onset of clinical symptoms. Several candidates have been proposed but all of these suffer from low sensitivity, in that not all PE patients are readily identified [4,7]. On the basis of the results described above, we hypothesized that previous poor biomarker performance [4] may have been due to the existence of these different subclasses of PE. To investigate this, we first assessed the expression of the two most frequently studied markers of preeclampsia, soluble FLT1 (sFLT1) and soluble ENG (sENG), produced in the placenta and found elevated in the maternal serum early in pregnancy [3]. The samples in the PE-enriched cluster 2 demonstrated increased expression of both of these common markers, while the remaining two clusters displayed much lower levels, in line with control values of expression (Fig. 4a).

Encouraged by this result, we next investigated the ability of PE markers to distinguish between the controls and the preeclamptic samples as a cohesive group, as well as split into PE subclasses. Using a subjective binary comparison employed by most typical analyses of this disease, we obtained a list of the top 10 genes with increased expression in the preeclamptics compared to the controls, all of which had been previously identified as potential markers of PE [8], including FLT1 and ENG (S4 Table). Visualization of the mean expression value of these 10 genes in control samples revealed a normal distribution (Fig. 4b). In contrast, the PE samples showed a higher mean expression and a bimodal distribution. When the mean expression was plotted for the preeclamptic placentas split into their three subclasses, the PE-enriched cluster 2 had the highest expression and was well separated from the controls, while the PE samples in clusters 1 and 3 displayed somewhat higher but strongly overlapping expression with the controls. These results indicate that through inclusion of all three subclasses of preeclamptics, the true gene expression differences are underestimated. Furthermore, using only the expression values of these 10 markers, Naive Bayes methods of classification and prediction was able to correctly separate more than 95% of the cluster 2 PE samples from the controls at a 5% false positive rate (FPR) (Fig. 4c). In contrast, only ~50% and ~40% of the preeclamptics in clusters 1 and 3, respectively, could be accurately categorized at this FPR. Combining all samples, these markers have a general ability to correctly identify 70% of all the PE samples as preeclamptic. Overall, these results indicate that current PE biomarkers very accurately identify cluster 2 preeclamptics, while the PE patients of subclasses 1 and 3 are not readily distinguishable from controls.

### Identification of novel PE molecular subclasses

**Fig 5:**
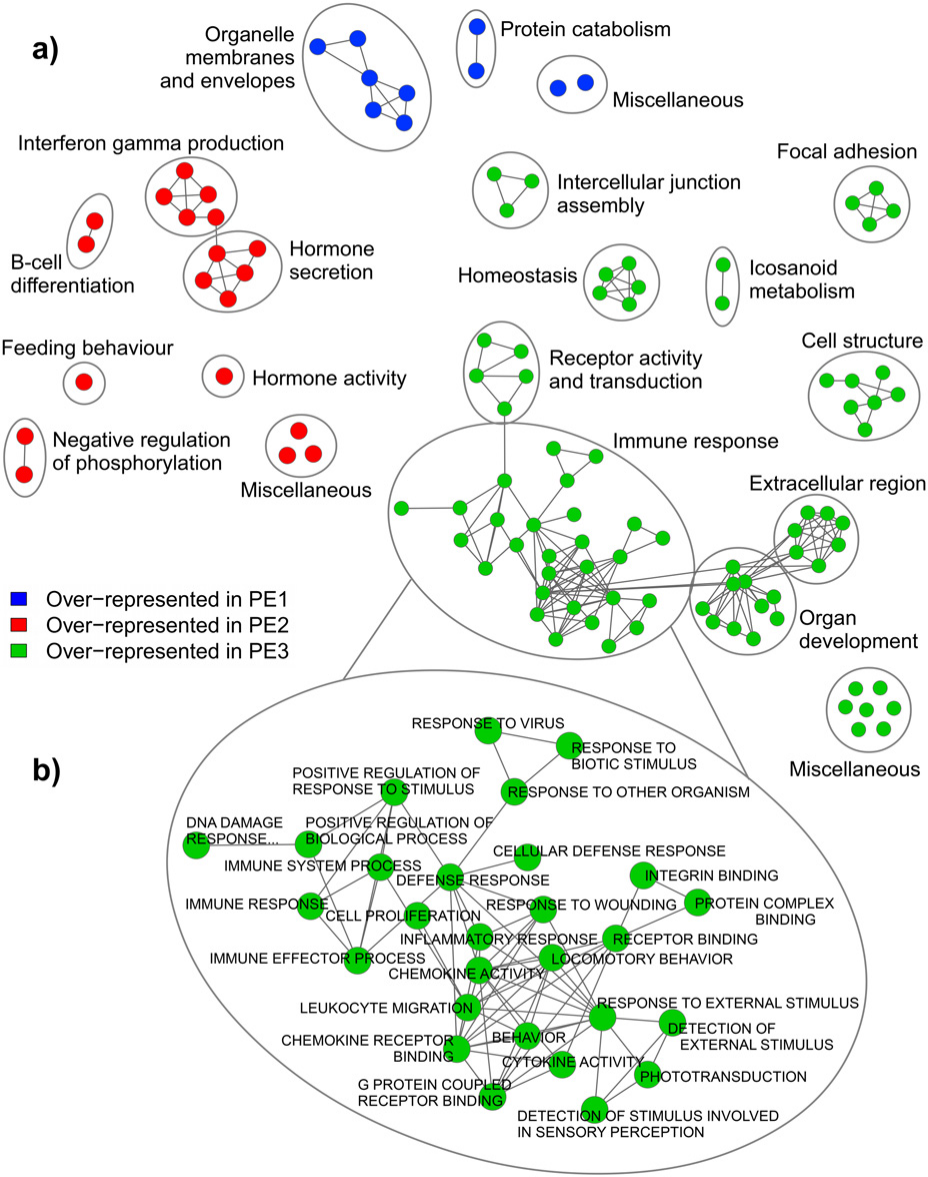
Gene set enrichment analysis (GSEA) results for the comparison of PE subclasses. GSEA outputs were visualized in Cytoscape and networks of related ontologies (shown as colored nodes connected by grey edges, representing common genes between gene sets) were circled and assigned a group label. Ontologies labeled as “miscellaneous” did not share genes with any of the networks. (A) In contrast to the remaining PE subclasses, the preeclamptics in cluster 1 (PE1) were found to be enriched in very few gene sets (blue), most of which were related to organelle membranes and envelopes; the preeclamptics in cluster 2 (PE2) displayed up-regulation of genes associated with feeding behaviour, B-cell activation, and hormone secretion (red); and the PE samples in cluster 3 (PE3) demonstrated an over-representation (green) of genes involved in organ development and extracellular matrix structure, as well as numerous terms associated with immune response. (B) Enlargement of the immune response network, including the *response to virus* ontology, enriched to cluster 3 PE samples with individual gene sets labelled. Overall, cluster 1 PE samples do not appear to demonstrate an overt PE pathology; the enrichments observed in cluster 2 PE samples fit with our canonical understanding of preeclampsia; and the PE samples in cluster 3 exhibit a potential pathogenic etiology of preeclampsia.

In the absence of detailed patient and placental data, we used gene set enrichment analysis (GSEA) to characterize the differences in molecular pathology between the three subclasses of PE patients (Fig. 5).

Compared to cluster 2 and cluster 3 PE samples, the preeclamptics in cluster 1 were found to be enriched in very few gene sets, most of which were related to organelle membranes and envelopes, as well as protein catabolism (Fig. 5, S5 Table). Down-regulated were ontologies involved in immune response, cell signalling, and tissue development and structure (S5 Table). Overall, these PE samples in cluster 1 do not demonstrate a significant enrichment in ontologies thought to be associated with a preeclamptic pathology, such as hypoxia and vascular development.

On the other hand, the preeclamptics in cluster 2 displayed an over-representation of genes involved in feeding behaviour, B-cell activation, interferon-gamma production, and hormone activity and secretion, as well as an under-representation of genes associated with oxidative phosphorylation (Fig. 5, S6 Table). Additional enrichments to this PE subclass were the hypoxia-inducible factor-1 (HIF-1) and-2 (HIF-2) pathways (S6 Table), which were largely driven by the up-regulation of known PE markers, such as ENG (HIF-1 pathway) and FLT1 (HIF-2 pathway). Together, this presents an injured tissue in a hypoxic environment, secreting increased amounts of placental products into the maternal compartment. These functional molecular phenotypes fit with the classical understanding of PE [28–31], which, along with the increased expression of known PE markers in this group (Fig. 4), diagnoses this subclass with “canonical” preeclampsia.

Cluster 3 PE samples demonstrated an up-regulation of genes involved in homeostasis, organ development, and extracellular matrix structure, as well as numerous terms associated with immune response, such as inflammatory response, defense response, cytokine activity, and response to wounding (Fig. 5, S7 Table). Further investigation also revealed an over-representation of genes linked to graft-versus-host disease and allograft rejection, which was driven, once again, by the up-regulation of HLA class II genes (S7 Table). Gene sets specific to the PE samples of cluster 3 were *DNA damage response signal transduction resulting in induction of apoptosis*, *response to other organism*, and *response to virus*, which imply a potential pathogenic source of placental damage, and perhaps even preeclampsia, in this subclass. Down-regulated ontologies were involved in female pregnancy, organelle function and membranes, and intracellular transport (S7 Table).

### Comparison of co-clustering controls and PE samples

Lastly, we decided to investigate whether the preeclamptic samples in clusters 1 and 3 could be separated from their co-clustered controls. Initial assessment of differential gene expression revealed very few genes (6 and 15 genes for clusters 1 and 3, respectively) reaching statistical significance (adjusted p-value < 0.01) in the preeclamptic samples compared to the control samples in both clusters (Table 3, S8 Table). This was in contrast to the large number of genes (nearly 3000) significantly differentially expressed in the cluster 2 PE placentas compared to both the cluster 1 and cluster 3 controls. Additionally, visualization of the mean expression of the top 5 genes up-regulated in the cluster 1 and cluster 3 preeclamptics compared to their co-clustered controls revealed significant overlap in expression between the two phenotypes (S3 Fig.), similar to the overlap observed with the known PE markers (Fig. 4b). Cluster 2 PE samples, on the other hand, demonstrated markedly superior separation from both subclasses of controls, likely due to greater fold-change differences in expression levels. This was further confirmed with Naive Bayes methods of classification and prediction which revealed that only ~60% of the cluster 1 and cluster 3 PE samples, compared to almost 100% of the cluster 2 PE samples, could be accurately segregated from controls at a 5% FPR using their respective set of top 5 differentially expressed genes (S3 Fig.). These results therefore imply that despite the existence of a few genes that achieve statistical significance, and have mild predictive power, between the preeclamptics and controls in clusters 1 and 3, these genes are not nearly as robust as cluster 2 PE markers, and are thus not sufficient for the identification of these non-canonical PE subclasses.

**Table 3:** The number of genes found to be up- and down-regulated in the preeclamptics of each cluster compared to the controls (adjusted p-value < 0.01).

	PE1 vs C1	PE3 vs C3	PE2 vs C1 and C3
Number of up-regulated genes	5	7	1327
Number of down-regulated genes	1	8	1661

It is possible that subtle, pathway-level gene expression differences exist between the PE samples and controls in clusters 1 and 3. In order to assess this, we compared the two phenotypes in each cluster by GSEA. No gene sets were found to be significant between the preeclamptics and controls in cluster 1 at a false discovery rate (FDR) of 25% (S9 Table). On the contrary, 8 gene sets were over-represented in a comparison of the PE and control samples of cluster 3 at this same FDR (S10 Table). This included *regulation of hormone secretion* and *feeding behaviour*, which are terms previously observed as enriched to the PE samples in cluster 2 (Fig. 5). However, as expected from the GSEA results described above (Fig. 3b, Fig. 5), the preeclamptics in cluster 3 also exhibited elevated expression of genes involved in the response to a virus, in contrast to controls. This indicates that while cluster 3 in general demonstrates an elevated immune response, the PE samples have an additional immune response, possibility a pathogenic cause of preeclampsia. The 20 significant genes annotated to this viral gene set are listed in Table 4.

**Table 4:** The list of 20 genes annotated to the GO ontology *response to virus* and found to be up-regulated in the preeclamptics of cluster 3 compared to their co-clustered controls.

Gene^a^	Protein Expression in Trophoblast^b^	Cell Component
ABCE1	High	Cytoplasm, membrane, mitochondria
**BNIP3**	Medium	Mitochondrial membrane
BNIP3L	Medium	Endoplasmic reticulum, mitochondrial membrane
CCL8	---	Secreted
CREBZF	None	Nucleus
**FGR**	Low	Plasma membrane
**IFI44**	Low	Cytoplasm
**IFNAR1**	Low	Plasma membrane
**IFNAR2**	High	Plasma membrane
IFNGR1	Medium	Plasma membrane
**IFNGR2**	Medium	Plasma membrane
IFNW1	---	Secreted
**IRF7**	High	Nucleus
ISG20	---	Nucleus
**PTPRC**	None	Plasma membrane
**RSAD2**	Low	Endoplasmic reticulum
SPACA3	None	Extracellular region, secretory granule, lysosome
**TLR8**	None	Membranes
**TNF**	---	Secreted
**TRIM22**	Medium	Cytoplasm

^a^The genes in bold were also enriched in comparison to the other PE subclasses.

^b^As detected by antibody staining of term placenta histology samples on Human Protein Atlas. A dashed line indicates that no trophoblast expression results were available for this gene.

## Discussion

We hypothesized that previously observed heterogeneity in preeclampsia, leading to a lack of robust predictive biomarkers and effective treatments for this disorder, was due to the existence of multiple molecular forms of PE. The considerable differences between these masked molecular groups have made the identification of biomarkers difficult, as there is low correlation between gene expression and the clinical symptoms of hypertension and proteinuria. Additionally, the failure of large scale randomized controlled trials [32,33] aimed at preventing PE was likely due to their treatment of these molecular subclasses as a single entity. To investigate this, we performed an aggregate analysis on 7 previously published PE microarray datasets, and clustered the samples based on gene expression alone, without accounting for clinical diagnosis. This unbiased approach led to the discovery of three patient clusters, all of which contained PE samples, thus supporting our hypothesis.

The surprising observation in our analysis was the discovery of both PE and control samples in clusters 1 and 3. The overarching question remains then: why are these PE patients and controls co-clustering? Genetically, these samples are grouping because they have very similar patterns of gene expression. Consequently, attempts at finding markers to separate the two phenotypes in each cluster identified very few genes with differential expression, while comparisons between clusters by GSEA unveiled comparable enrichments to the cluster 1 controls and PE samples (organelle membranes and function, and protein modification and activity) and to the cluster 3 controls and PE samples (extracellular matrix structure, immune response, and allograft rejection). We therefore propose the following potential explanations that, when combined, may account for this unexpected finding:

First, it is possible that some of the PE patients, particularly in cluster 1, may have been misdiagnosed as preeclamptic, and were really afflicted with another maternal hypertensive disorder, such as gestational hypertension or chronic hypertension [34]. This is supported by the GSEA and gestational age comparisons, which indicate that cluster 1 is largely composed of the healthiest term placentas in this data set. Furthermore, it is known that gestational hypertension does not cause the same increases in sENG and sFLT1 levels as PE [3,35], and that affected placentas are more histologically normal as compared to PE, specifically demonstrating lower fibrin deposits and syncytial knots [36]. Therefore, it is also anticipated that their placental gene expression would have more similarities with the healthy controls of cluster 1 than the “canonical” preeclamptics of cluster 2.

An additional explanation is poor or advantageous maternal adaptation to pregnancy. Pregnancy leads to many physiological changes in the mother [37], such as reduced vascular resistance and increased insulin resistance. A failure of the mother’s adaptive processes could result in the symptoms of PE despite a normal placenta, which would also explain the co-clustering of the cluster 1 PE samples with the healthy controls. The converse could also occur where the mother adapts to an abnormal placenta, reducing the severity of the symptoms and improving the outcome. This is likely the case for the controls in cluster 3, where an earlier poor placental event may have been resolved or compensated for by the maternal immune system but left a mark of increased immune response.

Lastly, and with the strongest argument for the phenotype mixture in cluster 3, is the likelihood that despite the aggregation of 7 microarray data sets, our final sample size of 173 may still be too underpowered to identify all existing clusters. Evidence for this explanation is the cluster 2 PE-related gene sets found to be significantly enriched to the PE samples in cluster 3 compared to their co-clustered controls. This overlap may be anticipated from the PCA plot of cluster membership as most of the PE cluster 3 samples are near the border of cluster 2 while the control samples are farther away. Therefore, a further increase in sample size may allow for cluster 3 to resolve into a control subgroup and a preeclampsia subgroup, demonstrating a mild but still existent PE phenotype. Additional support for this theory exists in the enrichment of viral response genes to the cluster 3 PE samples only.

Of the 20 significant genes annotated to this *response to virus* ontology, most are known to be expressed in the placental trophoblast based on Protein Atlas database records [38] (Table 4), and form a contiguous cellular pathway, spanning the plasma membrane, cytoplasm, and nucleus based on Entrez annotation [38,39]. The inclusion of 4 genes usually not expressed in healthy placentas may indicate either immune cell invasion or aberrant ectopic expression. Additionally, these 20 genes appear to be involved in a general viral response, associated with a range of different viruses, and not specific to any single infectious entity, based on Entrez annotation [39]. This indicates the possibility of a plurality of viral infection types occurring among the cluster 3 PE samples, such that responses to specific viruses are not apparent. Potential culprits are cytomegalovirus (CMV) [40], human papilloma virus (HPV) [41,42], and adeno-associated virus-2 (AAV-2) [43], as these are all known to be capable of infecting placental trophoblasts and have been observed to be associated with PE [42,44].

Furthermore, we found that cluster 3 samples demonstrated an over-representation of genes associated with allograft rejection and graft-versus-host disease (GVHD), compared to the samples in clusters 1 and 2. These ontologies have been previously linked to poor pregnancy outcome and the development of preeclampsia [45,46]. However, the majority of the significant genes annotated to these gene sets, including the HLA class II molecules, are not usually expressed in placental cells [47–49]. This enrichment is therefore likely due to an increased infiltration of maternal immune cells, which do express these genes, into the placenta. Although this may simply be a component of the GVHD response, maternal leukocyte infiltration can also occur in the placental response to a virus [50,51]. Additionally, viral infection and specific combinations of HLA isotypes have been shown to have compounding effects on pregnancy outcome, including preeclampsia development [52]. Therefore, while it is evident that the PE samples in cluster 3 demonstrate a heightened immune response relative to the remaining samples, it is unclear if this is a true viral infection or multiple, potentially compounded, immunologically regulated events. Regrettably, as we do not have direct access to alternate preparations of patient samples, it is not possible to investigate these theories, or any of our results, with histological examinations or targeted assays.

Regardless, our observation of multiple molecular PE subclasses is significant as it confirms that standard clinical tests for preeclampsia are not sufficient to distinguish these different groups. Specifically, we have demonstrated that current PE biomarkers are excellent at identifying cluster 2 patients, but are inadequate for the recognition of cluster 1 and cluster 3 PE samples. Additionally, each of these subclasses display different previously published phenotypes of PE: cluster 2 PE samples demonstrate an over-representation of genes associated with HIF signalling, and hormone production and secretion; PE samples of cluster 3 are enriched in genes related to immune response; and cluster 1 PE samples likely represent a poor maternal response to pregnancy that presents without overt placental pathology, a group that is often overlooked in the literature [10,53]. What is unique about our study is that these subclasses have clustered apart from each other, strongly indicating the existence of multiple causative sources of preeclampsia, and revealing molecular pathways that mark each group.

Unfortunately, the delivered placenta samples used in this analysis represent end stage disease and cannot directly identify the origin of pathology. This is a common issue to many diseased states, such as cancer, where the study of a tumour does not immediately inform the tumour-initiating event. Nevertheless, tumour analysis has led to great advances in identifying diagnostic and prognostic markers of cancer, and importantly, the identification of molecular heterogeneity of tumours has led to optimized treatment strategies. Discovering the true cause of a disease will require the creation of cellular and animal models, which are informed by unbiased genome wide analysis. Future work will determine if our observation of multiple outcomes (clusters) is really due to multiple initial causative insults or one insult that is modified by maternal and environmental agents to different end stages. Thus, while our results do not definitively solve PE, they do present a new path towards its solution using molecular approaches and individualized medicine.

Although the use of deposited and archived gene expression data is an excellent resource, our analysis also highlights the necessity of having detailed clinical records available for all human patient studies such that a more complete covariate examination can be performed and gene to phenotype relationships can be tested. Additionally, given the mild sampling bias observed within the control placentas, this work emphasizes the importance of obtaining multiple biopsies per placenta in order to control for the high degree of variability in gene expression frequently observed across the same tissue [25]. Finally, our study also indicates the necessity of having sufficient sample size in order to be able to distinguish biologically meaningful subgroups within a heterogeneous human population. While this is the largest data set of PE samples analyzed to date, it is highly probable that a further increase in placental number would identify additional clusters, likely demonstrating rarer but important pathological and physiological characteristics.

## Conclusion

Overall, our analysis represents a significant advancement towards understanding the underlying molecular heterogeneity of preeclampsia. Similar large-scale approaches could also be taken for other major pathologies of pregnancy, such as intra-uterine growth restriction and gestational diabetes myelitis. Moving forward, it will be essential to confirm and expand on these results with additional large-scale studies, an effort under way in our groups. Future emphasis should be on linking histopathology with gene expression data, and identifying and classifying patients early in diagnosis, likely through testing serum samples for panels of genes unique to each cluster. It is likely that current molecular diagnostics, such as sFLT1 and sENG, may efficiently select the “canonical” PE subclass. There is therefore a need to focus on the other two clusters, as placental gene expression cannot readily separate the preeclamptics from controls.

## Methods

### Study selection

Previously published preeclampsia-associated placental gene expression data sets were reviewed using a set of inclusion and exclusion criteria. Exclusion criteria were sampling at earlier stages of gestation (1st trimester), sampling of non-placental tissue, or sample set redundancy. While early sampling may reveal molecular origins, without longitudinal samples of the same patient over time, a correlation cannot be made between early gene expression patterns and end-stage expression patterns. Inclusion criteria were a minimum of three or more patient samples of a preeclamptic pathology (both early and late onset types), an array platform with more than 15,000 gene features, and the availability of raw data tables. A diagnosis of preeclampsia was defined as two or more episodes of hypertension (>140/90 mmHg) with proteinuria after the 20^th^ week of pregnancy. Proteinuria was not consistently defined between studies; values ranged from 300mg-2g of protein in a 24 hour period or >2+ on a dipstick test. As all the patient data was de-identified and obtained from previously published reports, the authors obtained an ethics waiver from the University of Toronto Office of Research Ethics.

### Assembly of the aggregate data set

The placental microarray data sets were loaded into R 3.0.1 from NCBI’s Gene Expression Omnibus (GEO) [54] using the *GEOquery* library. Gene expression values were extracted from each GEO series and converted into log2 intensities. For most arrays, the deposited annotation files could be used with the exception of three studies (GSE10588, GSE43942 and GSE4707) where the files were built using the *annotationforge* package from Bioconductor. The GSE25906 data set was batch corrected for the two indicated batches in the supplied annotation files, and 8 samples with fetal growth restriction (FGR) were removed from the GSE24129 data set. The individual sample sets were then aggregated into one array using the *virtualArray* package [15] (Bioconductor), which combines the compatible rows of the expression matrices and performs empirical Bayes methods of normalization and batch correction for the different original data sets. Finally, the merged data set was filtered for genes with expression variance in the top quartile. This cut-off was chosen to select for those genes with the highest potential information content for clustering patients.

### Clustering and co-variate analysis

The control and PE samples were treated as a single dataset and subjected to unsupervised multivariate model-based clustering, using the *mclust* [55] package from CRAN. The optimal number of clusters was selected based on the information obtained from the Bayesian Information Criterion. Principal component analysis (PCA) was performed on the transpose of the expression matrix, which allowed for the visualization of the clusters in component space using the *rgl* library. Information about the clinical phenotype (PE or control), gestational age (25–40 weeks; binned), nationality (Canada, China, Finland, Japan, or USA), and occurrence of labor (yes, no, or unknown) was added to the array for each sample. Fetal sex was typically not reported but was predicted based on the expression of two Y-chromosome genes: UTY and USP9Y. Chi-squared analysis was employed to test the significance of these patient variables on cluster membership. Lastly, principal variance component analysis (PVCA), using the *pvca* library, was performed on the full aggregate data set in order to determine the main sources of variability within the data.

### Investigation into the splitting of the control samples

Given that many of the original studies reported obtaining their placenta samples from a single biopsy, the possible existence of a sampling bias was investigated as a possible cause for the splitting of the controls. This was done by calculating the mean expression of 35 genes known to be significantly up-regulated in endothelial cells as well as the mean expression of 20 genes significantly enriched in trophoblasts, across each of the controls. A scaled (0 mean and 1 variance) heatmap of these mean expression values was then produced using the *heatmap* function and reversed heat colours such that the sample with the lowest mean endothelial-enriched or trophoblast-enriched expression value was coloured white and the sample with the highest value in each was coloured red. This was also done for each gene individually. Further inquiry into the splitting of the control samples was performed using the Molecular Signatures Database (MsigDB) collections associated with GSEA v2.1.0 [56] against a background model of the 14,653 genes found in common across all original microarray platforms. All C5 GO gene sets (v4.0) with 10–1000 members were assessed, which includes those annotated to Biological Process, Cellular Component, and Molecular Function, as well as C2 Canonical Pathways gene sets (v4.0), which includes KEGG, Protein Interaction Database, and Reactome collections, among others. The recommended number of permutations (1000) was performed using the less stringent (gene set) permutation type. GSEA GO results were visualized in Cytoscape v2.8.3 using the two-colour Enrichment Map plugin [57], with a p-value cutoff of 0.01, a corrected false discovery rate (FDR) q-value cutoff of 25%, and an overlap coefficient of 0.5. Finally, nodes were re-coloured to reflect the control subclass in question, and networks of related ontologies were circled and assigned a group label.

### Assessment of known PE markers

The differential expression of FLT1 and ENG was visualized with a 3-dimensional PCA plot, using colour gradients to demonstrate increasing expression of FLT1 (green to orange) and ENG (green to blue). Samples with low levels of both markers were therefore colored green, while those with elevated expression of both colored pink. A list of the top 10 genes with significantly increased expression in the PE samples compared to the controls was obtained from *limma* [58](Bioconductor). The mean expression of the 10 PE markers was calculated across each sample and a density plot of these values was produced using the *sm*.*density*.*compare* function from the *sm* library, split by phenotype as well as PE subclass. The WEKA machine learning software package [59] was then employed to evaluate the ability of these 10 genes to discriminate all PE samples from controls, and each PE subclass from controls, using a Naive Bayes classifier and 10-fold cross-validation. Marker performance was assessed by plots of the classifiers’ receiver operator characteristic (ROC) for the PE samples only.

### Identification of novel PE molecular subclasses

The three PE subclasses were compared to each other in a triangular fashion in GSEA, using the same settings described above in the assessment of the splitting of the controls, to produce three sets of GSEA results. Two out of three of these result sets were loaded into Cytoscape at a time, depending on which PE subclass was under investigation (ex. the PE1 vs PE2 results and the PE1 vs PE3 results were used to study enrichments to PE1). Two data set, two-colour enrichment maps were employed to simultaneously determine gene sets over- and under-represented in a given PE subclass compared to both remaining PE subclasses. Nodes were re-coloured to reflect the PE subclass in question, and networks of related ontologies were circled and assigned a group label.

### Comparison of co-clustering controls and PE samples

Lists of differentially expressed genes between the preeclamptics and controls in each of clusters 1 and 3 were obtained from *limma*, with an adjusted p-value cut-off of 0.01. The PE samples in cluster 2 were compared to both subclasses of controls simultaneously using *limma*, and only genes demonstrating adjusted p-values < 0.01 and the same direction of differential expression (either up- or down-regulated) in both comparisons were deemed significant. The mean expression of the top 5 genes up-regulated in each PE subclass, compared to either their co-clustered controls or all controls, was calculated across each sample involved in the original comparison and 3 density plots of these values were produced using the *sm*.*density*.*compare* function. The WEKA machine learning software package was also used to evaluate the ability of these three sets of 5 genes to discriminate the PE subclasses from either their co-clustered controls or all controls, using a Naive Bayes classifier and 10-fold cross-validation. Marker performance was assessed by ROC plots for the PE samples only. Additionally, GSEA was employed to investigate pathway-level differences between the co-clustering preeclamptics and controls, using the same settings described above in the assessment of the splitting of the controls. Placental trophoblast expression for each gene found to be significantly enriched to the *response to virus* GO ontology was assessed using Human Protein Atlas [38], and the cell component(s) of expression was/were determined from the information contained in NCBI’s Entrez Gene database [39].

## Supporting Information

S1 Fig Principal component analysis (PCA) of additional potential confounding factors of clustering. (PDF)

S2 Fig Full heatmap of expression of genes previously established as being enriched to placental trophoblast or endothelial cells in each of cluster 1 and cluster 3 controls. (PDF)

S3 Fig Density plots of the mean expression of, and receiver operator characteristic curves using, the top 5 genes significantly upregulated in each PE subclass compared to either their co-clustered controls or all controls. (PDF)

S1 Table Genes previously identified as enriched to either trophoblast or endothelial cells, used to investigate the possibility of a cell-specific sampling bias. (XLSX)

S2 Table Gene sets found to be significantly enriched to cluster 1 controls compared to cluster 3 controls by GSEA. (XLSX)

S3 Table Gene sets found to be significantly enriched to cluster 3 controls compared to cluster 1 controls by GSEA. (XLSX)

S4 Table Genes significantly upregulated in the PE samples compared to the controls. (XLSX)

S5 Table Gene sets found to be significantly enriched to cluster 1 PE samples compared to cluster 2 and cluster 3 PE samples by GSEA. (XLSX)

S6 Table Gene sets found to be significantly enriched to cluster 2 PE samples compared to cluster 1 and cluster 3 PE samples by GSEA. (XLSX)

S7 Table Gene sets found to be significantly enriched to cluster 3 PE samples compared to cluster 1 and cluster 2 PE samples by GSEA. (XLSX)

S8 Table Genes significantly upregulated in the PE subclasses compared to their co-clustered controls or all controls. (XLSX)

S9 Table Gene sets found to be moderately over-represented in cluster 1 PE samples compared to cluster 1 controls by GSEA. (XLSX)

S10 Table Gene sets found to be at least moderately over-represented in cluster 3 PE samples compared to cluster 3 controls by GSEA. (XLSX)

S11 Table Full aggregate data set with all covariates and normalized gene expression values for all 330 samples. (XLSX)
