# Supplementary material for: Large Scale Aggregate Microarray Analysis Reveals Three Distinct Molecular Subclasses of Human Preeclampsia
Source: PLoS One. 2015 Feb 13;10(2):e0116508. doi: 10.1371/journal.pone.0116508 (PMC4332506; doi:10.1371/journal.pone.0116508)
Supplement: S1 Fig — (PDF) [file pone.0116508.s001.pdf]

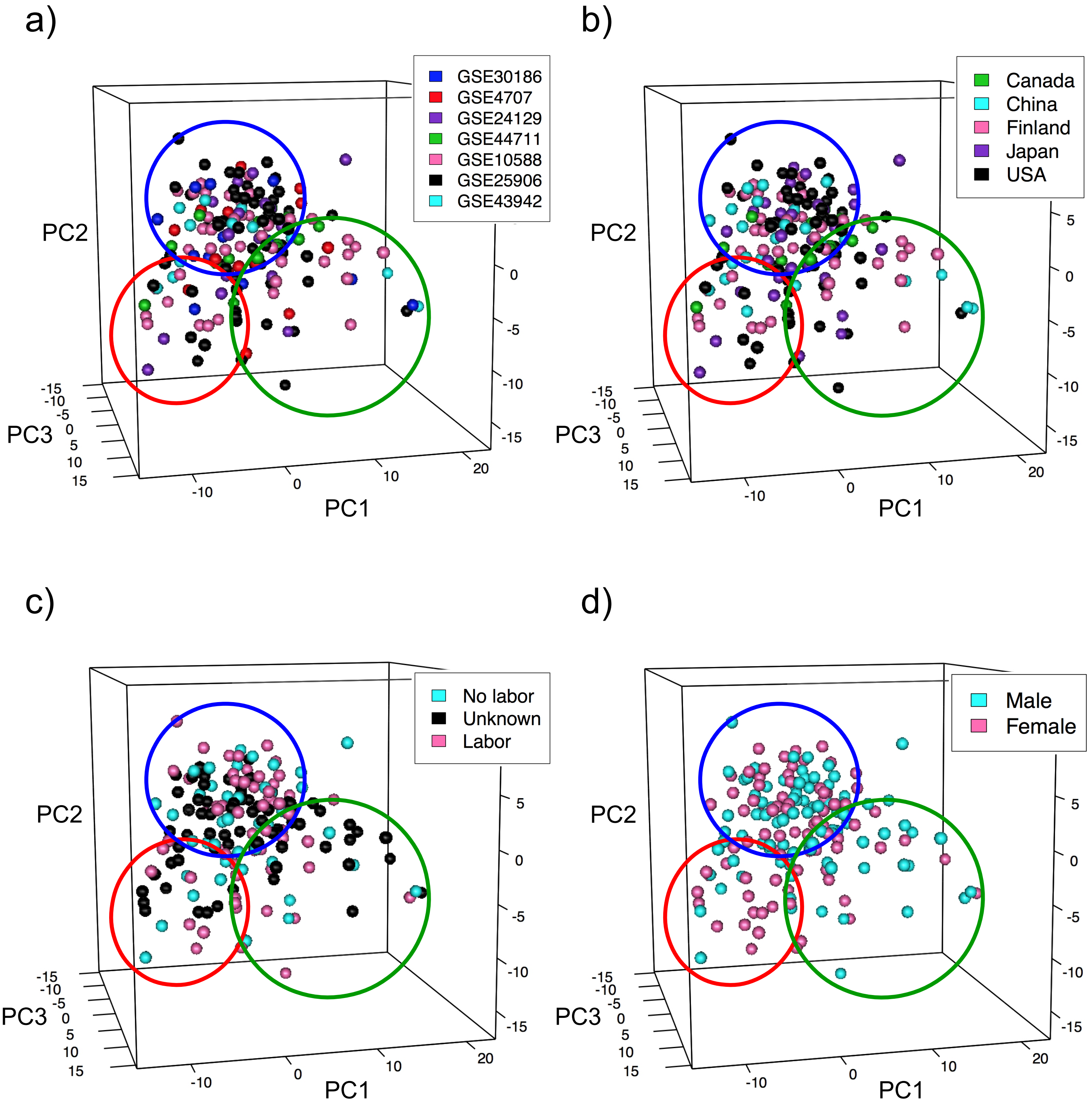

Supplementary Figure 1. Principal component analysis (PCA) of additional potential confounding factors of clustering. None of the other known covariates ((**a**) original study membership, (**b**) nationality, (**c**) occurrence of labor, or (**d**) fetal sex) demonstrated differential segregation between cluster 1 (circled in blue), cluster 2 (circled in red) and cluster 3 (circled in green). This observation was supported by chi-squared analysis (Table 2).
