# Supplementary material for: Large Scale Aggregate Microarray Analysis Reveals Three Distinct Molecular Subclasses of Human Preeclampsia
Source: PLoS One. 2015 Feb 13;10(2):e0116508. doi: 10.1371/journal.pone.0116508 (PMC4332506; doi:10.1371/journal.pone.0116508)
Supplement: S2 Fig — (PDF) [file pone.0116508.s002.pdf]

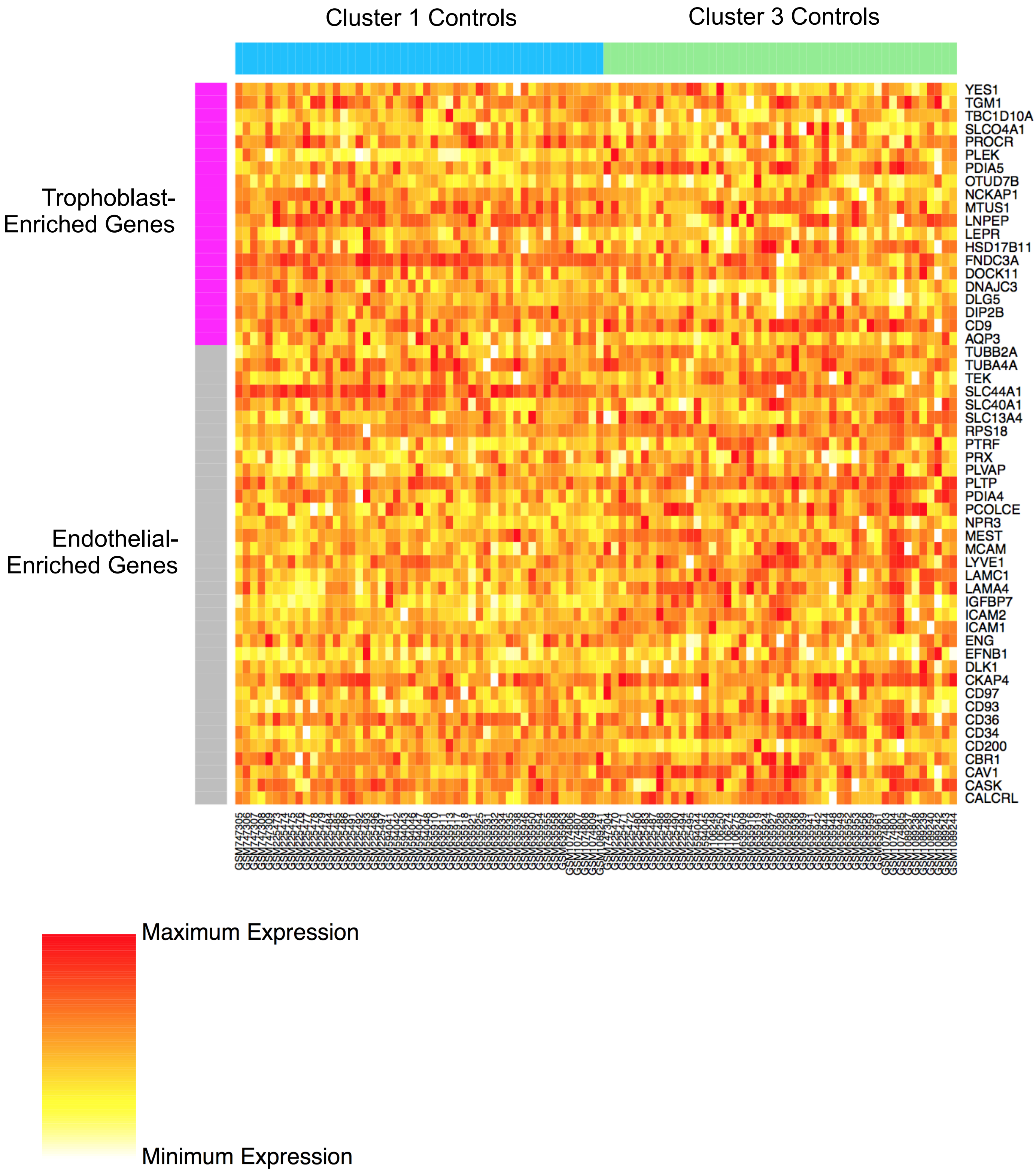

Supplementary Figure 2. Full heatmap of expression of genes previously established as being enriched to placental trophoblast (magenta) or endothelial (grey) cells in each of cluster 1 (blue) and cluster 3 (green) controls. Samples with high expression are colored red, with a gradient of decreasing expression down to white.
