# Supplementary material for: Large Scale Aggregate Microarray Analysis Reveals Three Distinct Molecular Subclasses of Human Preeclampsia
Source: PLoS One. 2015 Feb 13;10(2):e0116508. doi: 10.1371/journal.pone.0116508 (PMC4332506; doi:10.1371/journal.pone.0116508)
Supplement: S3 Fig — (PDF) [file pone.0116508.s003.pdf]

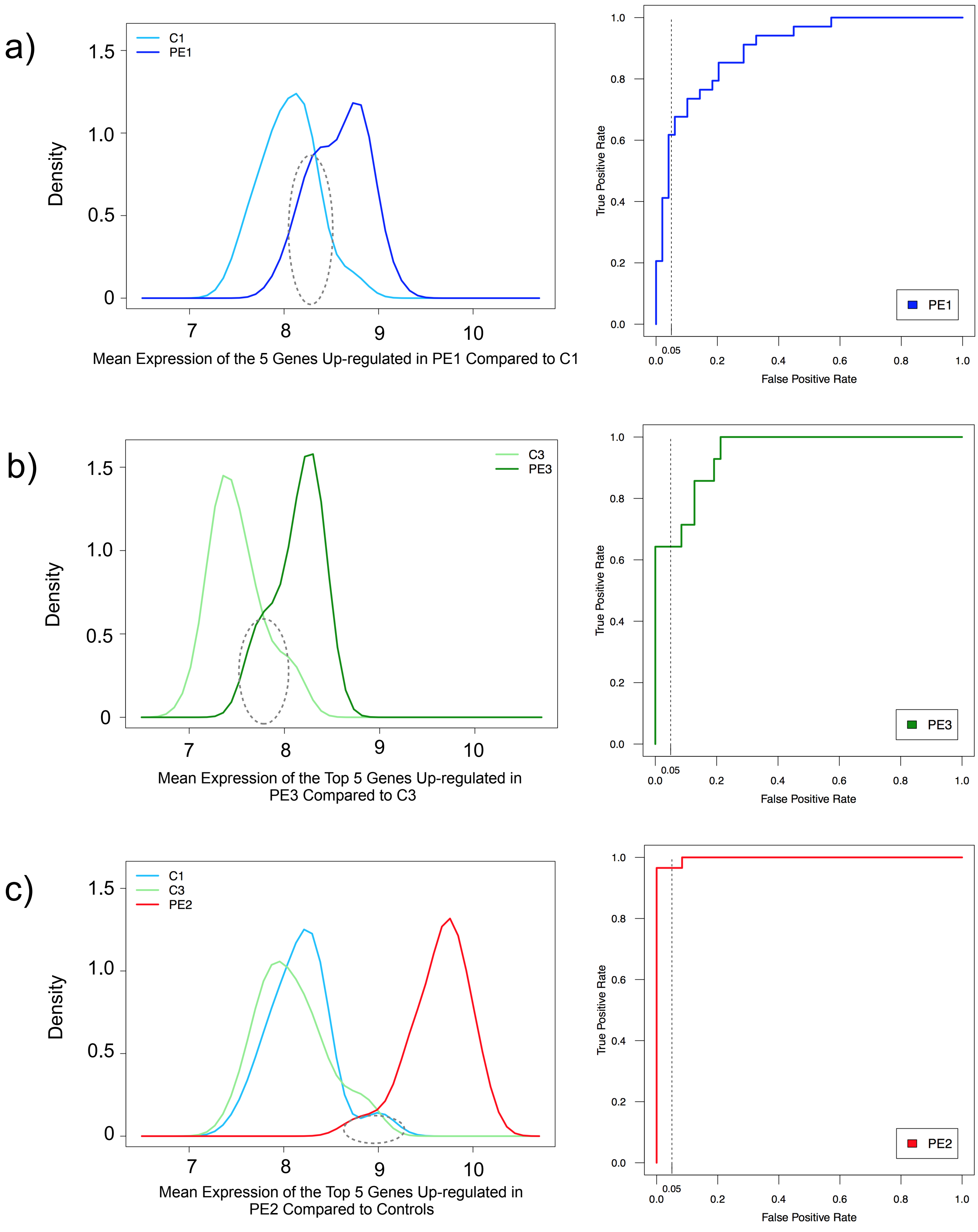

Supplementary Figure 3. Density plots of the mean expression of and receiver operator characteristic curves using (**a**) the only 5 genes significantly up-regulated in PE1 (dark blue) compared to C1 (light blue); (**b**) the top 5 (out of 7) genes up-regulated in PE3 (dark green) compared to C3 (light green); and (**c**) the top 5 (out of 1327) genes up-regulated in PE2 (red) compared to C1 and C3. The PE samples in cluster 2 can be much more easily predicted and separated from the controls than the cluster 1 and 3 PE samples, even with these top differentially expressed genes.
